# Restless legs syndrome or diabetic peripheral neuropathy: diagnostic dilemmas and clinical insights from a case report

**DOI:** 10.3389/fendo.2026.1899929

**Published:** 2026-08-04

**Authors:** Yongwen Zhang, Xiaoxi Nan, Lanfang Chu

**Affiliations:** 1 Department of Endocrinology, Nanjing Integrated Traditional Chinese and Western Medicine Hospital Affiliated with Nanjing University of Chinese Medicine, Nanjing, China; 2 Department of Gastroenterology, General Hospital of Eastern Theater Command, PLA, Nanjing, China

**Keywords:** diabetic peripheral neuropathy, differential diagnosis, pregabalin, restless legs syndrome, type 2 diabetes

## Abstract

**Objective:**

Diabetic peripheral neuropathy (DPN) comorbid with restless legs syndrome (RLS) is relatively rare and easily causes diagnostic dilemmas due to highly overlapping clinical manifestations. This study aimed to clarify the differential diagnosis and clinical recognition of DPN comorbid with RLS.

**Research design and methods:**

A retrospective analysis was conducted on a case of type 2 diabetes with lower extremity discomfort. We reviewed the prevalence of RLS in diabetic neuropathy, its diagnostic workflow, distinguishing features between RLS and DPN, as well as the evolving therapeutic concepts for RLS.

**Results:**

The patient presented with 6-month lower extremity discomfort, rest-induced aggravation, movement-related relief, nocturnal exacerbation, and severe sleep disturbance. Nerve conduction study and DPN screening were abnormal. Pregabalin therapy significantly alleviated symptoms. A diagnosis of secondary RLS was confirmed.

**Conclusions:**

Secondary RLS is easily confused with DPN. Strict guideline-based assessment and comorbidity management are critical for accurate diagnosis and optimal care.

## Introduction

Restless legs syndrome (RLS) constitutes a sleep-associated movement disorder, the core clinical feature being an irresistible urge to move one or both lower extremities, and occasionally the upper limbs, during periods of physical inactivity (1). This sensation is frequently accompanied by dysesthetic discomfort in the affected limbs, shows partial or complete remission upon motor activity, and typically exhibits diurnal variation with marked exacerbation during evening and nocturnal hours (1). In adult populations, differential diagnoses that may simulate RLS symptomatology include peripheral neuropathy, drug-induced akathisia, spasticity, positional limb discomfort, arthrogenous pain, and nocturnal leg cramps (1, 2). Given the absence of validated objective diagnostic biomarkers or laboratory assays for RLS, a meticulous and comprehensive clinical history remains indispensable for establishing an accurate diagnosis. Clinically relevant RLS, defined by symptomatic episodes at least twice weekly accompanied by at least moderate subjective distress, affects approximately 4.1% of adolescents (3). A significant association was observed between RLS and daily electronic device use for≥3 hours among boys (adjusted OR 1.63, 95% confidence interval [CI] 1.07-2.46), while girls faced elevated risks from <2 weekly moderate aerobic/lower-limb resistance sessions (≥30 min/session, 40%–59% heart rate reserve), delayed sleep onset, and menstrual abnormalities (dysmenorrhea, irregular cycles, oligomenorrhea, premenstrual dysphoric disorder) (3). Cyclic sex hormone shifts disrupt central dopamine signaling and iron utilization to worsen RLS. Importantly, these juvenile risk factors do not generalize to our 61-year-old male patient with longstanding type 2 diabetes and DPN-induced secondary late-onset RLS, whose clinical and risk profiles differ greatly from adolescents (3). Painless RLS was significantly correlated with a prior history of iron deficiency and systemic comorbidities, whereas painful RLS was associated with multiple chronic primary pain disorders (4). Findings from a study support a model in which circadian disruption promotes ferroptosis in RLS, contributing to iron overload, oxidative damage, and dopaminergic dysfunction (5). This ferroptosis model seems contradictory to systemic iron deficiency in painless RLS due to compartmentalized iron imbalance between periphery and central nervous system. Systemic iron shortage drives striatal dopaminergic neurons to absorb excess iron, causing intracellular labile iron buildup. Circadian disruption aggravates oxidative damage, explaining coexisting systemic iron depletion and neuronal iron overload in RLS patients (6). A cardinal morbidity of RLS is severe sleep disturbance, as nocturnal immobility becomes highly intolerable, resulting in insomnia. Approximately 90% of RLS patients report difficulties with sleep initiation and maintenance. Following sleep onset, patients with RLS commonly manifest periodic limb movements during sleep (PLMS) (7). On polysomnographic evaluation, PLMS present as brief, stereotyped flexion movements of the lower limbs, with a duration of 0.5–10 seconds and a recurrence interval of approximately 15–30 seconds. These movements predominate during the initial four hours of sleep and display substantial inter-night variability (4, 7). PLMS concentrate within the first four hours of sleep, largely confined to non-rapid eye movement (NREM) stage, rapid eye movement sleep-related global muscle atonia suppresses spinal motor activity and markedly reduces PLMS frequency, whereas partial motor excitability in NREM sleep facilitates these involuntary limb movements (8). A study indicates that leg subcutaneous adipose tissue may play a potential role in the pathophysiology of RLS, independent of BMI (9). Thinner mid-calf subcutaneous fat reduces peripheral iron and vitamin D storage, impairs lower-limb resting perfusion, and forms a vicious cycle with repetitive involuntary limb movements to worsen RLS sensory urges (9). Notably, thigh subcutaneous fat confers systemic metabolic advantages via adiponectin secretion and lipid buffering to mitigate insulin resistance, whereas calf subcutaneous fat only modulates local limb microenvironment without such metabolic protective effects, highlighting critical functional divergence between these two lower-extremity subcutaneous fat depots (9).

Diabetic peripheral neuropathy (DPN) is the most prevalent microvascular complication of diabetes, characterized by length-dependent mixed small- and large-fiber damage (10). Obesity and adipose dysfunction independently accelerate DPN progression via chronic low-grade inflammation and aggravated insulin resistance. Lower-extremity subcutaneous fat is closely associated with both DPN and RLS independent of BMI, which accounts for the high overlap of the two disorders in type 2 diabetic patients with metabolic dysfunction (11). One clinical study investigated the prevalence of RLS in patients with diabetic neuropathy and detected RLS in 33 of 99 individuals with neuropathy related to diabetes mellitus, impaired glucose tolerance, or impaired fasting glucose (12). Compared with patients without RLS, those with RLS more frequently presented with small-fiber sensory neuropathy (15/33 vs. 15/66) and burning foot symptoms (10/33 vs. 6/66) (12). A case-control study further identified polyneuropathy as the only independent factor linked to RLS in diabetic patients, with an odds ratio (OR) of 7.88 (95% CI 1.34–46.28; *P* < 0.02) (13). Such wide CI mainly stems from the limited sample size of this single case-control study and the relatively low number of participants with combined diabetic polyneuropathy and RLS. The clinical profile of RLS in diabetic patients is consistent with that of secondary RLS (13). The secondary RLS arising from DPN may also be driven by central nervous system alterations: sustained aberrant afferent signals following peripheral nerve injury can trigger a spectrum of central pathological changes, including spinal central sensitisation, impaired brainstem dopaminergic pathways and remodelling of the thalamocortical sensory circuit (14). However, current studies on the association and differential diagnosis between RLS and DPN remain limited. These two conditions frequently overlap in diabetic patients and pose substantial diagnostic challenges in clinical practice. This case report presents a typical patient with diagnostically ambiguous manifestations, aiming to explore the key points for the differential diagnosis of RLS and DPN, and to provide clinical references for accurate identification and management of similar cases.

## Case presentation

**Figure 1:**
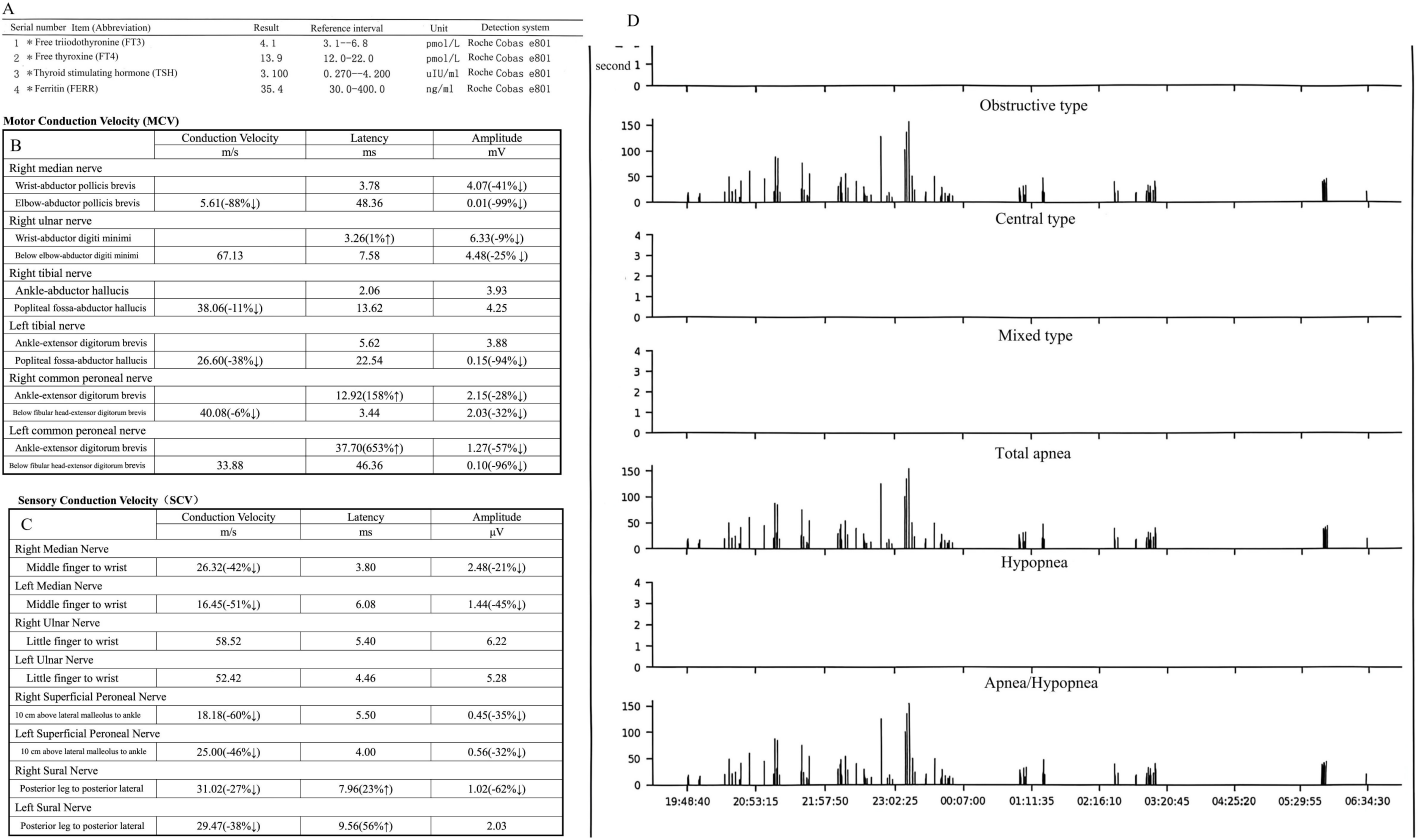
Clinical data of the patient: **(A)** Serum ferritin; **(B)** Motor conduction velocity, **(C)** Sensory conduction velocity, and **(D)** Polysomnography.

A 61-year-old male patient was admitted due to elevated blood glucose for more than 20 years and discomfort in both lower extremities for half a year. He had a 20-year history of diabetes mellitus and had been treated with insulin aspart 30 injection and acarbose for a long time, with poor glycemic control. Over the past six months, he developed a burning sensation and numbness in both lower extremities, accompanied by an irresistible urge to move the limbs repeatedly. Symptoms were transiently relieved by movement, aggravated at rest, and markedly exacerbated at night, severely impairing sleep. The patient had no history of hypertension or other chronic comorbidities. No hereditary or familial diseases were reported. No negative psychosocial factors or psychological disorders were identified during hospitalization. Initial screening for DPN was performed, including neurological examination (light touch, pinprick, 10g monofilament, 128Hz vibration test, and ankle tendon reflexes), and vibration perception threshold (VPT) measurement. Neurological sensory assessments were conducted on bilateral toe pulp, foot dorsum and anterior plantar area. Reduced sensation was confirmed when sensory thresholds were elevated or responses diminished (15). The neurological examination showed decreased distal sensation in the lower extremities, diminished ankle reflexes, and impaired vibration perception. After admission, laboratory examinations showed glycated hemoglobin (HbA1c) 8.1%, serum glucose 8.9 mmol/L, and serum ferritin 35.4 ng/ml (normal reference: 30–400 ng/ml) (**Figure 1A**). Ultrasound of the arteries and veins of bilateral lower extremities revealed no abnormalities. Nerve conduction velocity (NCV) study demonstrated prolonged distal motor latency, decreased amplitude, and slowed conduction velocity in bilateral common peroneal and tibial nerves; sensory nerve conduction testing showed reduced conduction velocities, prolonged latencies and decreased amplitudes in bilateral median, superficial peroneal and sural nerves, while bilateral ulnar nerve parameters remained normal (**Figures 1B, C**). Polysomnography indicated mild obstructive sleep apnea-hypopnea syndrome (**Figure 1D**). Following dose adjustment of insulin aspart 30 injection and addition of dapagliflozin tablets, glycemic control was satisfactory (fasting blood-glucose 6.0-7.2 mmol/L, and 2-hour postprandial blood-glucose 7.8-8.9 mmol/L). No overly rapid glycemic correction was performed, and aggravation of neuropathic symptoms secondary to treatment-induced neuropathy was not observed in our patient. However, the lower extremity discomfort showed no obvious improvement after treatment with mecobalamin and epalrestat. The patient still complained nocturnal exacerbation and temporary relief after activity, with severe sleep disturbance and frequent sleepless nights. Administration of zolpidem tartrate tablets 10 mg at bedtime failed to improve sleep. The patient had painful DPN and painful RLS, manifesting burning foot pain, circadian worsening and urge to move limbs with dysesthesia. After an in-department difficult case discussion, some clinicians supported a diagnosis of secondary RLS, others emphasized DPN, and still others advocated a combined diagnosis of DPN and RLS, with no consensus reached. Since both conditions are responsive to pregabalin, pregabalin tablets were added at a dose of 75 mg twice daily. Two days later, the patient’s symptoms were markedly relieved and sleep was improved. The patient was discharged in improved condition. The patient was followed up for six months after discharge, with stable clinical conditions and no recurrent restless legs syndrome symptoms.

## Discussion

RLS exhibits a significantly higher prevalence in patients with hereditary neuropathy, whereas no such association is evident in those with acquired neuropathies (16). A pooling analysis of observational studies also demonstrated that diabetic patients carry an increased risk of RLS compared with non-diabetic subjects (OR 1.98, 95%CI 1.66–2.34, *P* < 0.001) (17). However, studies on the diagnostic distinction and integrated treatment of DPN combined with RLS are still limited, highlighting an urgent need for more evidence in this comorbid population.

**Figure 2:**
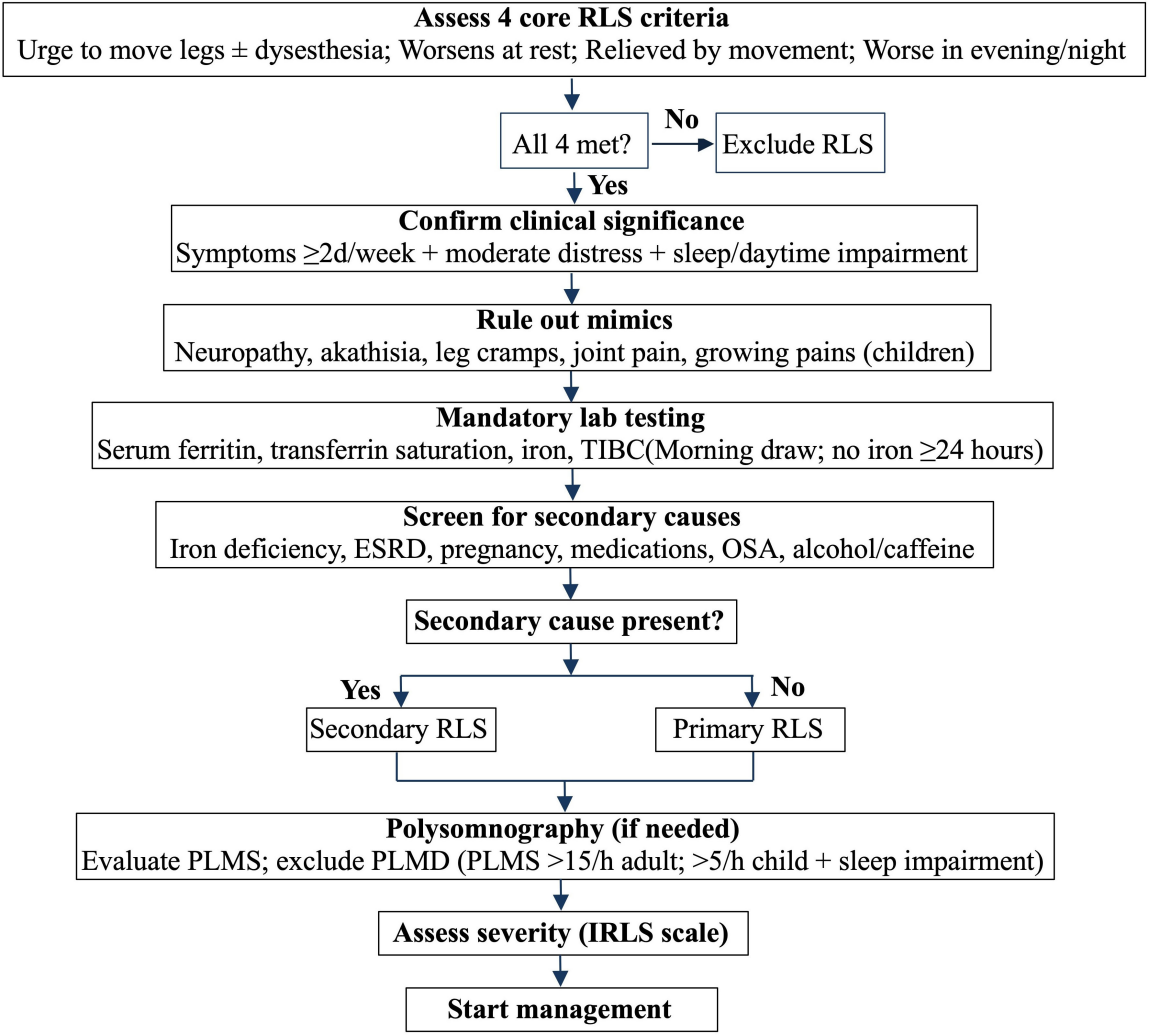
RLS diagnostic flowchart (AASM 2025) TIBC, total iron binding capacity; ESRD, end-stage renal disease; OSA, obstructive sleep apnea; PLMS, periodic limb movements in sleep; PLMD, periodic limb movement disorder.

This American Academy of Sleep Medicine (AASM) 2025 RLS diagnostic checklist outlines a concise clinical workflow (**Figure 2**) (18). Diagnosis relies on four mandatory criteria: the urge to move limbs with dysesthesia, worsening at rest, relief with movement, and circadian worsening. Symptom frequency, distress level, and impact on sleep/daytime function confirm clinical significance. Iron deficiency, pregnancy, medications, obstructive sleep apnea (OSA), alcohol, and other mimics must be excluded first. All patients undergo mandatory iron metabolism testing (ferritin, transferrin saturation [TSAT], iron, total iron binding capacity [TIBC]) under standardized conditions. Polysomnography is optional for PLMS/periodic limb movement disorder (PLMD) detection. Severity is assessed via the IRLS Scale, with special protocols for children, pregnancy, and end-stage renal disease (ESRD) populations (18).

For this patient, motor nerve predominance in electrophysiological lesions is an atypical but documented manifestation of DPN rather than exclusive to proximal peripheral nerve lesions (19), the differential diagnoses include secondary RLS, DPN, or DPN comorbid with RLS, even after expert discussion, no consensus had been reached regarding the patient’s diagnoses. Distinguishing between DPN and RLS relies on marked differences in clinical presentation, pathological mechanisms, and therapeutic strategies (**Table 1**). In terms of symptomatology, DPN is characterized by persistent sensory deficits and neuropathic pain, whereas RLS manifests as paroxysmal sensory urges that are characteristically aggravated by rest and alleviated by motor activity. Pathologically, DPN represents a primary peripheral neuropathy involving distal axonal injury, while RLS is fundamentally a central nervous system disorder mainly associated with central iron deficiency, in which peripheral small-fiber alterations occur only as secondary changes. Correspondingly, therapeutic management differs substantially: interventions for DPN emphasize glycemic control and the alleviation of peripheral neuropathic pain, whereas RLS treatment prioritizes the correction of central iron deficiency and the modulation of central dopaminergic function and neuronal excitability (18–21). These distinctions are consistent with the 2025 AASM clinical practice guidelines for RLS/PLMD and the established clinical features of DPN as reported in the relevant literature (**Table 1**). According to the diagnostic algorithm of RLS and the differential diagnosis between DPN and RLS, this patient presented with abnormal NCV, abnormal initial screening results for DPN, and typical clinical manifestations of RLS. Therefore, a diagnosis of secondary RLS was confirmed. Such a diagnosis emphasizes that, in addition to the treatment of RLS, attention should also be paid to the management of DPN, including glycemic control, assessment of risk factors, prevention of foot ulcers and amputations, and symptomatic pain management.

**Table 1:** Comparison of DPN and RLS in pathogenesis, therapeutic recommendations and prognosis.

Dimension	DPN	RLS
Clinical Symptoms	Positive symptoms (pain, dysesthesia): Burning, lancinating, stabbing, tingling, allodynia (10).	Sensory Urge: Uncomfortable creeping, crawling, or aching sensation in legs (18).
	Negative symptoms (sensory loss): Numbness, loss of vibration/position sense.	Worse at Rest: Inactivity (sitting/lying) exacerbates symptoms; relieved by movement.
	Distribution: Distal-to-proximal (glove-stocking), worse at night.	Circadian Rhythm: Peaks in the evening/night, disrupting sleep.
	Motor: Weakness (late-stage, large-fiber involvement) (19–21).	Motor: Involuntary leg movements (PLMD) during sleep (22).
Nerve Fiber Involvement	Mixed (Primary Small, Secondary Large) Early: Small fibers, pain and temperature sensation loss. Progression: Large fibers, vibration/proprioception loss and ataxia. Typical: Mixed presentation in most patients (21).	Predominantly Small-Fiber (Central Etiology)
		Periphery: Possible small-fiber loss, but not the primary cause.
		Center: Core pathology involves low brain iron and central dopaminergic dysfunction (23).
Etiology and Pathogenesis	Microvascular and Metabolic Damage Chronic hyperglycemia: oxidative stress and AGE accumulation. Microvascular ischemia and Chronic inflammation (TNF-α, IL-1β) and Schwann cell damage. Length-dependent degeneration: Longest nerves (distal extremities) affected first (24).	Multifactorial (Central and Peripheral) Iron deficiency: Reduced brain iron (ferritin), dysregulation of dopaminergic pathways. Genetic predisposition (PRRT2, BTBD9 genes). Peripheral sensory neuropathy (secondary finding). Dysregulation of spinal cord excitability and hypoxic pathways (22).
First-Line Treatment	Glycemic Control + Symptomatic Pain Management α2δ ligands: Pregabalin, Gabapentin (first-line for neuropathic pain); SNRIs: Duloxetine, Venlafaxine; Topical agents (lidocaine patch) for focal pain (10).	Iron Repletion + Dopaminergic/α2δ Ligands IV Iron: Ferric carboxymaltose (strong recommendation for low ferritin); α2δ ligands: Pregabalin, Gabapentin (first-line); Dopaminergics: Levodopa (short-term only; high risk of augmentation) (18).
Key Diagnostic Signs	Physical Exam: Decreased pinprick/temperature (small fiber), decreased 128Hz vibration, diminished ankle reflexes.	Clinical Diagnostic Criteria (AASM):Urge to move legs with discomfort; Worse with rest; Relieved by movement; Worse evening/night.
	Testing: Normal NCV (early small-fiber), abnormal NCV (late large-fiber), reduced skin biopsy IENFD.	Testing: Polysomnography (PLM index), serum ferritin/TSAT.
Prognosis and Outcomes	Progressive: Peripheral sensory loss leads to high risk of foot ulcers, and amputation.	Chronic but Non-Degenerative: No direct nerve degeneration or limb loss.
	Irreversible: Nerve damage is often chronic; treatment slows progression (21).	Treatable: Iron repletion and medication significantly improve sleep quality and quality of life (24).

The 2012 AASM clinical practice guideline (CPG) for RLS management identified pramipexole and ropinirole as standard therapeutic agents. Levodopa combined with a dopa decarboxylase inhibitor, opioids, gabapentin enacarbil, and cabergoline were classified as guideline-level recommendations with corresponding precautions (25). Multiple clinical trials and long-term observational studies have further clarified the application of RLS-targeted medications, as reflected in the latest RLS treatment guidelines issued by other academic institutions (26). In particular, the long-term risk of augmentation, an iatrogenic exacerbation of RLS symptoms related to dopamine agonists, has been better delineated, leading the clinical practice guideline to highlight augmentation as a key outcome and re-evaluate the risk-benefit profile of this drug class (27). Augmentation is characterized by progressive worsening of RLS symptom severity and duration after months to years of dopaminergic exposure, presenting as earlier symptom onset, shortened latency at rest, and/or symptom extension to other body regions. Additionally, augmentation is more frequent and severe at higher dosages, and clinical dose increments often result in non-linear progression of symptom intensity (18).

According to the 2016 American Academy of Neurology guideline, pharmacotherapy should be considered for moderate-to-severe primary RLS (28). Pramipexole, rotigotine, cabergoline, and gabapentin enacarbil are supported by high-level evidence; ropinirole, pregabalin, and intravenous (IV) ferric carboxymaltose are backed by moderate evidence; whereas levodopa is regarded as an optional intervention. Currently, limited head-to-head clinical trials are available to guide preferential drug selection. Prolonged-release oxycodone/naloxone may be considered for refractory cases (Level C). Iron supplementation with ferrous sulfate plus vitamin C is recommended for RLS patients with serum ferritin ≤75 μg/L (Level B).Among nonpharmacological strategies, pneumatic compression is advised (Level B), while near-infrared irradiation, transcranial magnetic stimulation, and vibrating pads may be used to alleviate symptoms or improve sleep quality (Level C).For hemodialysis patients with secondary RLS, combined vitamin C and E therapy is recommended (Level B), and ropinirole, levodopa, or exercise training may be implemented as adjunctive measures (Level C) (28).

In 2025, the AASM issued strong recommendations favoring gabapentin enacarbil, gabapentin, pregabalin, or IV ferric carboxymaltose for adult patients with RLS, supported by moderate-certainty evidence. Conversely, conditional recommendations were issued against routine use of levodopa, pramipexole, transdermal rotigotine, ropinirole, bupropion, carbamazepine, clonazepam, valerian, valproic acid, or cabergoline, based on very low to moderate evidence certainty (18). For all individuals with clinically relevant RLS, serial assessment of serum iron markers, including ferritin and transferrin saturation (derived from serum iron and total iron-binding capacity), is strongly advised. Iron panel results play a pivotal role in determining whether oral or IV iron supplementation is indicated. Primary management of RLS should begin with identification and modification of potential exacerbating factors, including alcohol, caffeine, antihistamines, serotonergic agents, antidopaminergic drugs, and untreated OSA (18).

The therapeutic approach to RLS has evolved significantly over the past decade. Early management centered on dopamine agonists and levodopa as first-line agents, yet long-term application revealed high risks of augmentation and other adverse reactions, prompting reassessment of their safety profile. With updated evidence, the 2025 AASM guideline redefined standard care: α2δ ligands (pregabalin, gabapentin) and IV iron are now preferred first-line treatments. Modern strategies prioritize evaluating secondary causes, correcting iron deficiency, and integrating comorbidity management, marking a shift toward safer, more individualized, and guideline-directed therapy.

When managing RLS secondary to DPN, clinicians should prioritize integrated treatment strategies that simultaneously target the underlying pathological alterations of DPN itself, rather than merely alleviating RLS-related limb discomfort. No disease-modifying drugs are available to reverse DPN, and therapeutic strategies have shifted from simple glycemic control to comprehensive metabolic management combined with stepped pain relief (10). Current guidelines prioritize weight loss, regular exercise, lipid and blood pressure regulation to relieve peripheral mitochondrial dysfunction and axonal damage. Rapid blood glucose correction should be avoided to prevent treatment-induced neuropathy. For painful DPN, a tiered analgesic regimen is recommended. First-line options include gabapentinoids, serotonin-norepinephrine reuptake inhibitors, tricyclic antidepressants and topical capsaicin with comparable efficacy (21). Opioids only offer short-term pain relief but carry high risks of dependence and organ toxicity, so long-term use is strongly discouraged. Spinal cord stimulation remains an unproven alternative for intractable cases (21). Novel therapeutic directions include sodium-glucose cotransporter-2 (SGLT-2) inhibitors, which show promising neuroprotective effects in animal studies but lack sufficient human clinical evidence (29). Our case report of the patient receiving dapagliflozin treatment serves as a direct clinical counterpart to the newly proposed therapeutic direction of SGLT-2 inhibitor application (30). Glucagon-like peptide-1 receptor agonists show neuroprotective and analgesic effects, exerting multi-target benefits against shared pathological drivers of neuropathic pain and neurodegenerative disorders (31). Non-pharmacological approaches like exercise and cognitive behavioral therapy serve as auxiliary measures to ease small-fiber injury and neuropathic discomfort. Major challenges include inconsistent diagnostic criteria, insufficient sensitive early biomarkers and slow translation of preclinical findings into disease-modifying therapies, calling for unified evaluation standards and novel targeted treatments (10).

This case report has several strengths: it documents a rare clinical presentation of RLS secondary to DPN; systematically collates the standardized diagnostic workflow for RLS, and elaborates the evolving therapeutic strategies for RLS based on updated clinical guidelines and high-quality references. However, several limitations should be acknowledged. The single-case design restricts generalizability; the presented electrophysiological manifestations (more severe motor nerve impairment than sensory nerve lesions) represent an atypical DPN phenotype, and pregabalin administration could be confounded by its dual efficacy against both neuropathic pain and RLS. Future prospective cohort studies with larger sample sizes and standardized multi-case comparative data are required to further validate our diagnostic conclusions and related pathophysiological mechanisms.

## Conclusion

This patient had a 20-year history of poorly controlled diabetes mellitus, accompanied by numbness, burning, and nocturnal leg discomfort. Clinical and electrophysiological findings supported DPN, while typical rest-aggravated, movement-relieved symptoms and sleep disturbance indicated RLS. A diagnosis of secondary RLS was confirmed. Although pregabalin effectively relieved symptoms, it could not clarify the etiology. Clinicians should strictly follow the AASM 2025 diagnostic criteria and differential workflow, perform comprehensive assessments including iron metabolism and polysomnography, confirm the diagnosis accurately, and implement guideline-directed therapy rather than empirical treatment.

## Data Availability

The raw data supporting the conclusions of this article will be made available by the authors, without undue reservation.
